# Evaluation of Surgical Outcomes of Type A Intramural Hematoma

**DOI:** 10.21470/1678-9741-2020-0223

**Published:** 2022

**Authors:** Tugra Gencpinar, Reha Topak, Ozkan Alatas, Aytac Gulcu, Serdar Bayrak, Cenk Erdal

**Affiliations:** 1 Department of Cardiovascular Surgery, Dokuz Eylul University, Faculty of Medicine, Izmir, Turkey.; 2 Department of Radiology, Dokuz Eylul University, Faculty of Medicine, Izmir, Turkey.

**Keywords:** Cardiopulmonary Bypass, Bicuspid, Aneurysm, Dissecting, Aorta, Hematoma, Prognosis, Hypertension, Intensive Care Units

## Abstract

**Introduction:**

In this study, we aimed to retrospectively evaluate the results of type A intramural hematoma (TA-IMH) cases that underwent ascending aortic surgery.

**Methods:**

One hundred ninety-four patients who underwent aortic surgery between 2010 and 2018 were included in this study. TA-IMH was differentiated according to tomography angiographic images. Demographic data, operation type, hypothermic circulatory arrest times, echocardiographic findings, wall thickness of IMH, complications, and prognosis were retrospectively analyzed.

**Results:**

TA-IMH (n=14) or type A aortic dissection (AD) (n=35) data were collected from patients’ files and 49 cases were enrolled into the study. Bentall operation was performed in eight patients (type A AD = six [17.1%], TA-IMH = two [14.3%]); 41 patients underwent tubular graft interposition of ascending aorta (AD = 29 [82.9%], TA-IMH = 12 [85.7%]). There was no significant difference in terms of age, gender distribution, aortic dimensions, cardiopulmonary bypass times, hypothermic circulatory arrest times, hospital ward stay, and intensive care unit stay between the two groups. The mortality rate of AD group was 34.4% and of TA-IMH group was 14.3%. There was no significant difference in terms of mortality between the groups. In our study, 45.7% of patients had hypertension and that rate was lower than the one found in the literature. In addition, bicuspid aorta was not observed in both groups. Connective tissue disease was not detected in any group.

**Conclusion:**

Surgical treatment of aorta is beneficial for TA-IMH. Our aortic surgical indications comply with the European aortic surgical guidelines. Hypertension control should be provided aggressively.

**Table:** 

Abbreviations, acronyms & symbols				
**AD**	**= Aortic dissection**		**ICU**	**= Intensive care unit**
**CPB**	**= Cardiopulmonary bypass**		**IMH**	**= Intramural hematoma**
**CT**	**= Computed tomography**		**PAU**	**= Penetrating atherosclerotic ulcer**
**ECMO**	**= Extracorporeal membrane oxygenation**		**SD**	**= Standard deviation**
**IABP**	**= Intraaortic balloon pump**		**TA-IMH**	**= Type A intramural hematoma**

## INTRODUCTION

Aortic intramural hematoma (IMH) was described for the first time by Krukenberg without intimal rupture or penetrating ulcer rupture of adventitial vasa vasorum and with bleeding in the subadventional area^[1]^. Acute aortic dissection (AD), IMH, and penetrating atherosclerotic ulcer (PAU) lesions in the Stanford type A distribution are pathophysiologies that can present high mortality and morbidity, which often require urgent open surgical repair. Clinically, differential diagnosis of IMH and AD is difficult. However, with current advanced diagnostic imaging systems, IMH can be distinguished from AD. Type A intramural hematoma (TA-IMH) is a potentially lethal condition under the heading of acute aortic syndromes^[1-4]^. Although there is hemorrhage to the aortic media layer in both IMH and AD, there is no intimal flap rupture and false lumen formation in the IMH. And even though it is not fully elucidated, the pathogenesis of TA-IMH is thought to be due to vasa vasorum rupture secondary to the presence of penetrating atherosclerotic ulcers. Type A AD and TA-IMH are similar in terms of risk factors and clinical complications, such as aortic aneurism, hemothorax, cardiac tamponade, or malperfusion. However, in the Stanford type B lesions, it may be treated medically in the absence of symptoms. In addition, a low threshold for endovascular or urgent surgical treatment should be maintained.

Due to our lack of knowledge about the natural history of the disease, there is no general consensus on TA-IMH management^[3-7]^. Therefore, the popular management approach in the Western countries is the immediate surgical repair of the aorta, while medical management in the Far East countries is primarily aimed, and surgical management is provided under special conditions^[3,4,8,9]^. The optimal treatment approach has not been established.

Our clinical experience is to evaluate TA-IMH and AD and to perform emergency surgical repair. In this study, we aimed to investigate the early results of TA-IMH patients who underwent surgical repair in our clinic.

## METHODS

Patients’ perioperative demographic data were recorded after obtaining permission of the ethics committee for non-interventional research of Dokuz Eylül University (decision date and number: 2018/2906). Data of 194 cases, who underwent aortic surgery between January 2010 and December 2018, were collected and analyzed retrospectively. Forty-nine cases with TA-IMH and/or AD diagnoses were enrolled into the study.

### Inclusion-Exclusion Criteria

Patients who underwent aortic surgery were included in the study. TA-IMH was defined by the absence of an intimal tear in computed tomography (CT) angiography. Patients who had elective aortic valve repair and replacement were excluded. Unstable patients whose neurological examination could not be performed because of cardiac arrest were excluded from the study. Radiologists diagnosed the development of cerebral malperfusion. Reoperation for the aorta and Marfan syndrome patients were excluded. Patients whose radiological imaging could not be found in the hospital database or whose data were missing were not included in the study.

### Patient Population

The diagnosis was made with contrast CT angiography. The indication of surgery for TA-IMH included the presence of symptoms, rapid dilatation of the aorta (> 10 mm/year), an ascending aorta > 55 mm, and/or thickness of a hematoma in the false lumen > 5 mm. The patients were divided into two groups as typical type A AD (AD group) (n=35) and TA-IMH (n=14). Age, gender, ascending aorta diameter, IMH size, operation type, hypothermic circulatory arrest time, cardiopulmonary bypass time, intensive care unit (ICU) length of stay, hospital ward stay, presence of bicuspid aorta, hypertension, diabetes, and mortality data were collected retrospectively. Table 1 shows demographic data and study parameters.

**Table 1:** Demographic data and study parameters.

		Type A Aortic Dissection (n=35)	TA-IMH (n=14)	*P*-value
**Demographic data**				
Age (mean±SD)		57.83±10.89	58.64±15.44	0.859
Gender, n (%)	Female	10 (28.6%)	5 (35.7%)	0.735
	Male	25 (71.4%)	9 (64.3%)	
**Comorbidities**				
Malignancy		1 (2.8%)	-	
Bicuspid aorta		-	-	
Hypertension		16 (45.7%)	5 (35.7%)	
Diabetes mellitus		1 (2.8%)	-	
**Radiological parameters**				
Aortic dimension (mean±SD)		53.64±7.96	54.64±9.78	0.382
Dimension of IMH (mean±SD)		-	18.43±8.23	
**Intraoperative parameters**				
Hypothermic circulatory arrest times (minutes, mean±SD)		125.60±55.42	101.64±37.17	0.215
CPB times (minutes, mean±SD)		212.43±111.21	165.57±50.87	0.138
**Postoperative parameters**				
ICU length of stay (days, mean±SD)		6.69±10.69	6.29±6.41	0.480
Hospital length of stay (days, mean±SD)		12.97±13.59	12.93±8.93	0.488
Mortality, n (%)		12 (34.3%)	2 (14.3%)	0.294
**Postoperative complications**				
Ulnar nerve ınjury		1 (2.8%)	-	
Pneumonia		2 (5.6%)	2 (14.3%)	
ECMO		1 (2.8%)	2 (14.3%)	
Transient ischemic attack		1 (2.8%)	-	
Post-perfusion syndrome		3 (8.4%)	-	
Pneumothorax		1 (2.8%)	-	
Reoperation		3 (8.4%)	3 (21.45%)	
IABP		2 (5.6%)	2 (14.3%)	
Acute renal ınjury		3 (8.4%)	2 (14.3%)	
Hemodialysis		1 (2.8%)	1 (7.15%)	

CPB=cardiopulmonary bypass; ECMO=extracorporeal membrane oxygenation; IABP=intra-aortic balloon pump; ICU=intensive care unit; IMH=intramural hematoma; SD=standard deviation; TA-IMH=type A intramural hematoma

**Fig. 1:**
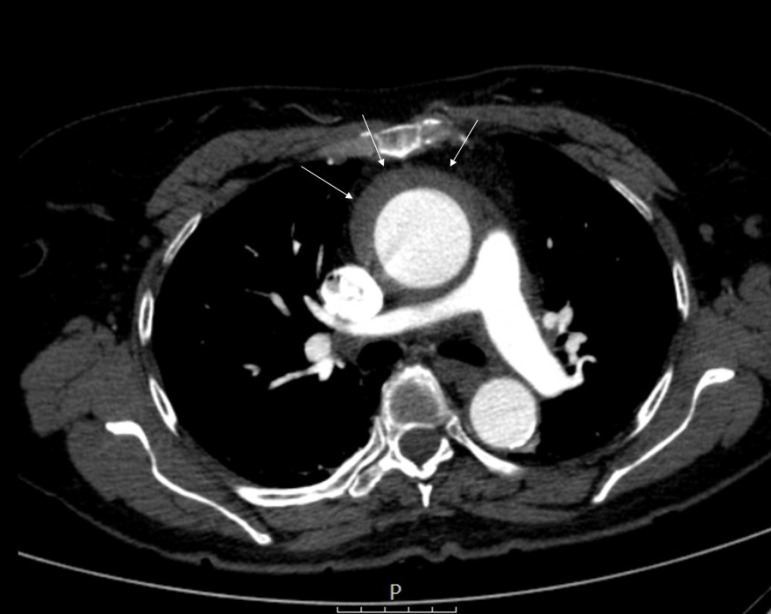
Computed tomography angiographic image showing type A intramural hematoma.

**Fig. 2:**
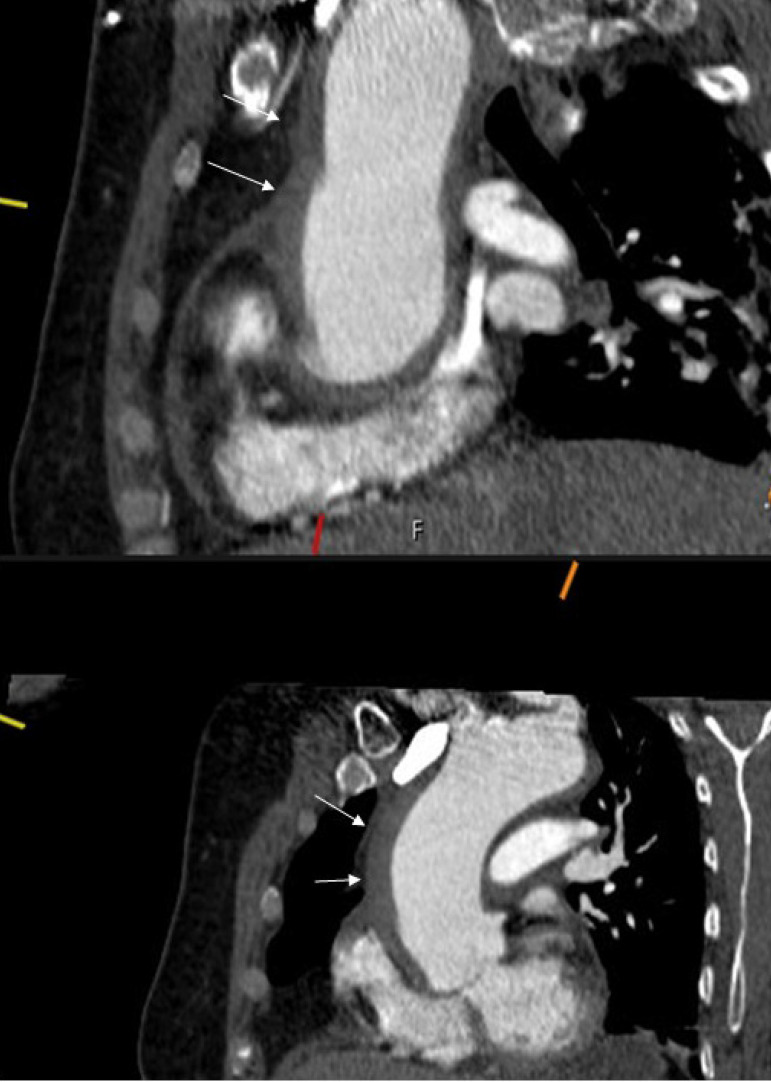
Computed tomography angiographic images showing type A intramural hematoma.

Aortic IMH was defined as the presence of a circular thickening in the aortic wall (≥ 5 mm) and a clinical coexistence consistent with acute aortic syndrome (Figures 1 and 2). CT angiographic images were examined by two radiologists. Ascending aorta, the largest diameters, and IMH dimensions were examined.

The same team performed all surgical procedures. Heat monitoring was done with nasopharyngeal and rectal probes. Patients managed surgically were operated via a median sternotomy, using cardiopulmonary bypass, and profound 28 °C hypothermic circulatory arrest. Autotransfusion system was not used at that moment. Patients were put in Trendelenburg position and deep hypothermic total circulatory arrest was initiated. Total circulatory arrest, deep hypothermic aortic intermittent clamping, and topical cooling myocardial protection were used in all cases. In addition, slow cooling and rewarming, avoidance of hypo/hyperglycemia, and hemodilution were used for cerebral preservation of total circulatory arrest application. Also, we performed optimal surgical management to avoid total circulatory arrest period > 45 minutes for cerebral protection. In order to prevent air embolism from entering the carotid arteries, the brachiocephalic truncus and left carotid artery were clamped during arrest. The patients were cooled down to 20 °C for total circulatory arrest. To prevent the development of no-reflow phenomenon in the brain and provide an adequate peripheral circulation, blood hematocrit was targeted as 20% at 20 °C. Blood pressure was monitored via online radial arteries. Selective anterograde cerebral perfusion was applied. We use antegrade cerebral perfusion selectively in aortic pathologies that are thought to prolong the circulatory arrest period. A 10-mm Dacron vascular graft was anastomosed to brachiocephalic or axillary artery for cardiopulmonary bypass and antegrade cerebral perfusion. Antegrade perfusion of the brain was then initiated while the circulatory arrest continues through this Dacron graft with blood from the artery line of the pump. Perfusion pressure was monitored with the radial artery cannula inserted into both arms and kept at an average of 50 mmHg. Selective antegrade cerebral perfusion pressure was applied as 10 ml/kg/minute. Brain protection was provided by anesthesia with the addition of steroids. Arterial cannulation was established via femoral artery, axillary artery, or ascending aorta. For typical AD, obliteration of the false lumen was performed with resection of the tear if feasible. For IMH, evacuation of all hematoma from the false lumen with subsequent obliteration was performed. Distal anastomosis without clamping on the aorta was only possible with total circulatory arrest. Distal anastomoses of the aorta were repaired with double Teflon felt. Proximal anastomosis of aorta was performed while rewarming up to 30°C.

### Statistical Analysis

Data were presented as mean and standard deviation (SD) or as numbers and percentages. Parametric and non-parametric tests were applied to the data according to their distribution characteristics. Chi-square test was used to compare the presence of bicuspid aorta, diabetes, hypertension, and mortality. All calculations and statistical analyzes were performed using IBM Corp. Released 2011, IBM SPSS Statistics for Windows, Version 20.0, Armonk, NY: IBM Corp. and Microsoft Excel (2011). *P* value < 0.05 was considered as significant.

## RESULTS

The mean age in the AD group was 57.83±10.89 years and in the TA-IMH group it was 58.64±15.44 years (SD = 12 years, range = 32-82 years); 69.4% (n=34) of the patients were male. Female and male ratios were 28.6 and 71.4%, respectively, and these were 35.7% and 64.3% in the TA-IMH group. There was no significant difference in terms of gender distribution between the groups.

When the evaluation was made in terms of comorbidities, it was observed that malignancy was present in only one patient (2.8%) in the AD group and no malignancy was observed in the TA-IMH group. Bicuspid aorta was not observed in any patient in both the AD and TA-IMH groups. Although hypertension was observed in 16 (45.7%) patients in the AD group and in five patients (35.7%) in the TA-IMH group, there was no significant difference between the groups (*P*=0.523).

In the preoperative CT angiograms, the mean diameter of ascending aorta from the widest section was measured as 53.64±7.96 mm in the type A AD group and 54.64±9.78 mm in the TA-IMH group, and no statistically significant difference was observed (*P*=0.382). The average IMH dimensions were 18.43±8.23 mm in the measurements made from the widest section.

Bentall operation (reconstruction of the aortic root) was performed in eight patients due to aortic root involvement (type A AD = six patients [17.1%], TA-IMH = two patients [14.3%]); 41 patients underwent tubular graft interposition from the ascending aorta (AD group = 29 patients [82.9%], TA-IMH = 12 patients [85.7%]). The mean cardiopulmonary bypass time was 212.43±111.21 minutes in the type A AD group and 165.57±50.87 minutes in the TA-IMH group; there was no significant difference between the groups (*P*=0.138). The mean hypothermic circulatory arrest time was 125.60±55.42 minutes in the AD group and 101.64±37.17 minutes in the TA-IMH group; there was no significant difference between them (*P*=0.215). Six patients (17.1%) in the AD group and two patients (14.3%) in the TA-IMH group were Penn class Aa (circulatory collapse); 29 patients (82.9%) in the AD group and 12 patients (85.7%) in the TA-IMH group were Penn class Ab (malperfusion with ischaemia).

Mean ICU length of stay was 6.69±10.6 days in the AD group and 6.29±6.4 days in the TA-IMH group, and no statistically significant difference was observed between the groups (*P*=0.480). However, the average length of hospital ward stay was 12.97±13.59 days in the AD group and 12.93±8.93 days in the TA-IMH group, and no statistically significant difference was observed between them (*P*=0.488). In-hospital mortality was observed in 12 patients (34.3%) in the AD group and two patients (14.3%) in the TA-IMH group. There was no statistically significant difference between the groups in terms of early postoperative mortality rates (*P*=0.294).

The need for reoperation as a result of tamponade and hemorrhage was found in six patients, transient neurological complication in one patient, renal failure in five patients, need for hemodialysis in two patients, and nosocomial pneumonia in two patients. Sepsis and mediastinitis were not observed. Extracorporeal membrane oxygenation was used in three patients and intra-aortic balloon pump in four patients as a result of left heart failure. As a neurological complication, transient ischemic attack was observed in only one patient (2.8%) with type A AD. Agitation and disorientation due to post-perfusion syndrome in three (8.4%) patients improved without sequelae in a short time. In addition, no other neurological results were seen.

Twenty-eight patients (48%) were Penn class Aa (absence of branch vessel malperfusion or circulatory collapse), 11 cases (19%) were Penn class Ab (branch vessel malperfusion with ischaemia), five cases (9%) were Penn class Ac (circulatory collapse with or without cardiac involvement), and 14 cases (24%) were Penn class Abc (both branch vessel malperfusion and circulatory collapse). The number of patients with localized or generalized ischaemia or both, Penn class non-Aa, was 30 (52%).

## DISCUSSION

Acute AD and IMH are pathologies associated with high mortality and morbidity. IMH classification and treatment are similar worldwide. However, there are some different recommendations in the United States of America, Europe, and Asia guidelines and such a consensus cannot be achieved for TA-IMH. In this study, 49 patients underwent ascending aorta surgery (Bentall operation or tubular graft interposition of ascending aorta) and were evaluated retrospectively. In accordance with the literature, the mortality rate of surgical repair of IMH was found as 14.3%. In CT angiography, the mean diameter of ascending aorta from the widest section was measured and no statistically significant difference was observed between the groups (*P*=0.382). In addition, no significant difference was found between the two groups in terms of age, gender distribution, aortic dimensions, cardiopulmonary bypass times, hypothermic circulatory arrest times, and in-hospital and ICU length of stay.

Approximately 10-20% patients with acute AD may not have a blood flow or intimal tear in the false lumen^[1-4]^. It is estimated that IMH is caused by bleeding of the vasa vasorum in the aortic media layer or by microscopic damage of the intima^[1-6]^. Aortic wall thickness is associated with the presence of acute aortic syndrome^[7-11]^. As a result of interventional procedures, iatrogenic IMH may develop at a rate of 0.04-0.8%^[12-13]^. IMH is gradually resorbed in long term or it may develop into AD. Preoperative malperfusion is important for the evaluation of patients with acute aortic type A AD and IMH type A. In this study, in-hospital mortality was not statistically significant different in Penn class Ab patients. We applied Penn classification, but we did not find a significant difference in mortality between the groups.

In addition, the evidence is limited by the inadequacy of randomized trials. Management of IMH is still controversial. In some Asian studies, mortality rates up to 8% have been reported in the early surgical repair of IMH^[5-11]^. Also there are poor outcomes of medical management of IMH with mortality rates ranging from 33% to 80%^[3-6,12]^. However, the absorption of the IMH have reported more than 60% and five-year survival is between 80-85% in some series^[5,7,10]^. A meta-analysis study, involving 12 studies with nine of them originated from Asian cohorts for the evaluation of the surgical and medical management of IMH, reported that there is no difference in terms of mortality between surgical repair or medical follow-up^[13]^. The mortality rate with the surgical repair of IMH was found as 14.3%. The studies in the literature evaluating the differences of aortic dimensions between TA-IMH and type A AD reported that there was no difference between them^[1,14,15]^. This study results are also compatible with them.

In a Japanese and Korean series^[10-12]^, 124 patients were followed up with 7% hospital mortality and remodeling was observed as a result of medical follow-up. CT performed patient’s follow-up for catastrophic complication and aneurysm. Song et al.^[7]^, in their series, reported that 24 (67%) of 36 proximal hematoma cases were resorbed. Nine (25%) of them had AD. Fifty-four (78%) of 69 patients with distal hematoma underwent resorption. Eleven patients (16%) progressed to AD. Three-year results are 78-87% in proximal and distal types^[7]^.

Ferrera et al.^[16]^, in a patient series with 23 type A IMH patients (57.5%) and 17 type B IMH patients (42.5%), reported that mortality was decreased dramatically in the acute phase due to reabsorption. In addition, they showed that mortality is associated with the acute stage of the disease. It had been followed that type A IMH continues in nearly 35.7% of the patients. They emphasized that hypertension control should be effective. Schoenhoff et al.^[17]^ reported that type A IMH open surgery was required in 89% of 63 IMH cases. Aorta-associated mortality rate was determined at 30 days, six months, and one year and reported as 1.6%, 6.3%, and 9.5%, respectively. Frequent follow-up should be performed. Conservative series of echocardiographic imaging is recommended^[17]^. Ganaha et al.^[18]^ emphasized that it is important to do early surgical treatment of type A IMH. They found that the amount of hematoma increases the wall stress, and this negatively affects the wall mechanical stability. In addition, they concluded that there are useful results supported by previous experimental studies. Also, as a result of models, increased blood clot can lead to more stable IMH^[18]^. They modeled that a small permeable hematoma was formed in high wall stress.

The prevalence of hypertension in patients with AD has been reported to be around 80% in the literature^[14-18]^. In this study, 45.7% of the patients operated for acute aortic diseases had hypertension. Hypertension control was achieved with beta-blocker agents in all patients. Intravenous nitrate treatment for hypertension control was used in ICU. Due to the presence of undiagnosed hypertension patients, the rate of hypertension was lower in our study than in the literature. In addition, bicuspid aorta was not observed in both type A AD and TA-IMH groups.

### Limitations

These findings could not be systematically confirmed by a pathological study of the aorta due to the absence of pathological specimens.

## CONCLUSION

Our study showed that TA-IMH is evaluated as acute AD. Classification and treatment should be evaluated by similar emergency surgical indication for acute AD. As stated in the literature, hypertension is often associated with the etiology of IMH. In order to prevent catastrophic complications of IMH, aggressive blood pressure control is greatly important.

**Table:** 

Authors' roles & responsibilities	
TG	Substantial contributions to the conception or design of the work; or the acquisition, analysis, or interpretation of data for the work
RT	Drafting the work or revising it critically for important intellectual content
OA	Agreement to be accountable for all aspects of the work in ensuring that questions related to the accuracy or integrity of any part of the work are appropriately investigated and resolved
AG	Final approval of the version to be published
SB	Drafting the work or revising it critically for important intellectual content
CE	Substantial contributions to the conception or design of the work; or the acquisition, analysis, or interpretation of data for the work
